# How long can you store vitamins? Stability of tocopherols and tocotrienol during different storage conditions in broccoli and blueberries

**DOI:** 10.1016/j.fochx.2024.101444

**Published:** 2024-05-06

**Authors:** Irmela Sarvan, Anton Jürgensen, Matthias Greiner, Oliver Lindtner

**Affiliations:** German Federal Institute for Risk Assessment (BfR), Max-Dohrn-Straße 8-10, 10589 Berlin, Germany

**Keywords:** Storage stability, Tocochromanoles, Broccoli, Blueberries, Frozen, Freeze-dried, Vitamin E

## Abstract

Differences between the stability of α-, β-, γ-, and δ-tocopherol as well α-tocotrienol stored at −20 °C and −80 °C were studied in broccoli and blueberry samples. Before storage up to 28 days, they underwent different initializing processes such as freezing quickly with liquid nitrogen and freeze-drying, followed by homogenization. While α-tocopherol levels in blueberries did not significantly differ, levels in broccoli were substantially higher after homogenization of freeze-dried samples compared to fresh broccoli samples. This might be caused by higher extractability of α-tocopherol from the changed cell structure. Storage of fresh broccoli samples at −20 °C led to decreasing α-tocopherol levels. Nevertheless, the deviation between freeze-dried samples to the initial fresh samples and fresh samples frozen with liquid nitrogen stored at −20 °C for 7 days were in the same order of magnitude. In conclusion, storage up to 7 days for vitamin relevant samples before analysis seemed to be justifiable.

## Highlights

• Blueberries: α-tocopherol levels consistent over 28 days at −20 °C.
• Broccoli: α-tocopherol levels decreased over 28 days at −20 °C.
• Higher α-tocopherol levels in freeze-dried compared to fresh broccoli.

## 1 Introduction

Tocopherols and tocotrienols, also cumulated called tocochromanols or vitamin E (Knecht, Sandfuchs, Kulling, & Bunzel, 2015; Zaaboul & Liu, 2022), are known to have antioxidative and anti-inflammatory properties (Shahidi & Ambigaipalan, 2015; Szewczyk, Chojnacka, & Górnicka, 2021). In the right dose, they are beneficial to the human health by protecting cells from the damaging effect of free radicals such as peroxyl radicals (Niki, 2014). In foods, they are rich in vegetable oils such as sunflower and wheat germ oil and other parts of higher plants (α-tocopherol especially in green parts of plants) (Shahidi & Ambigaipalan, 2015). Besides their natural origin in foods, tocochromanols are also used to prevent peroxidation of fats and lipids by the food industry (EFSA ANS Panel, 2015).

Tocochromanols are differentiated into α-, β-, γ-, and δ-tocopherol as well α-tocotrienol, depending on the number and/or positions of methyl groups at the chromanol ring (Zaaboul & Liu, 2022). After ingestion, α-tocopherol is released into the bloodstream by the α-tocopherol transfer protein. Because other forms of tocochromanols are recognized poorly by the transfer protein and cannot be converted into α-tocopherol in the human body, α-tocopherol has the highest bioavailability (Jiang, 2022) and is often used as single factor for vitamin E requirements and vitamin E intake recommendations (IOM (Institute of Medicine), 2001; EFSA 2015). In contrast, the German Nutrition Society (DGE) uses tocopherol equivalents in their recommendations (DGE (Deutsche Gesellschaft für Ernährung e.V., The German Nutrition Society), 2024).

Besides its health beneficial properties, too high levels in our diet might lead to increased liver weight and reduced blood clotting (EFSA, 2015). To assess beneficial or potentially harmful effects to our health, it is necessary to know the exact level of substances in our food (Sarvan, Bürgelt, Lindtner, & Greiner, 2017). Nevertheless, estimation of levels is only possible, if the effect of storage conditions of samples till the analysis is known and taken into account. Depending on the storage conditions, levels of tocochromanols might change. In the presence of oxygen, light or high temperatures, the levels of tocochromanols in food were found to decrease (Bergström, 1994). Furthermore, the pH of the foods may influence the storage stability as well.

In literature, studies on tocochromanols were conducted mainly as stabilizers or antioxidants for other food components and not on the stability of tocopherol itself. To our knowledge, no systematic study was done on tocochromanol stability in fruit and vegetable with different storage conditions.

Levels of vitamins such as K1, K2, β-carotene, folate and tocochromanols are analyzed in pooled samples of the first German Total Diet Study, called BfR MEAL Study (“Mahlzeiten für die Expositionsschätzung und Analytik von Lebensmitteln“, meals for exposure estimation and analysis of foods) (Schendel et al., 2022). Foods are prepared as the consumer would do before eating, including not only cooking or steaming, but also e.g., removing the core end eventually the skin of apples. Thereafter the ready-to-eat samples are combined with similar foods to one pool and are homogenized, before sending the samples to the appropriate laboratories for the analysis. The generated data on levels of substances in foods will be matched with average consumption data of the German population and the oral exposure to these substances will be assessed (e.g., Sarvan et al., 2021). To assure there are no differences between measured concentrations in the laboratories compared to the initial prepared and homogenized sample of the BfR MEAL Study, the storage conditions and maximum storage time of samples before analysis had to be defined. Tocochromanols were defined as the most instable vitamin of all vitamins measured in the BfR MEAL study and therefore should be used as indicator for the storage stability.

The aim of this research was to determine the effect of different homogenization conditions (fresh with liquid nitrogen or freeze-dried) and storage temperature (at −20 °C or − 80 °C) on tocochromanol levels in blueberries and broccoli. We selected broccoli and blueberries as representative foods with different pH levels. The knowledge gained in this research was used to develop a method for handling foodstuffs prior vitamin analysis. This method should be standardized and reduced the degradation of vitamins before analysis as far as possible. Furthermore, it could help us to assess the ratio between the analyzed values and the values present in the food.

## 2 Material and methods

### 2.1 Sample preparation

Broccoli and blueberries were bought in different supermarkets in Berlin, Germany, in 2016. Stems and leaves of the blueberries were discharged and the berries mixed carefully, without damaging the structure of the berries. Broccoli was cut into florets containing 2 cm stem and mixed by hand thoroughly. At least 2.5 kg of each homogenous material was freeze-dried and blended in a mixer (Grindomix GM300, Retsch GmbH, Haan, Germany). 500 g of the same fresh material was frozen with liquid nitrogen and blended (Grindomix GM300, Retsch GmbH, Haan, Germany). Half of the material of each grinding condition was stored at −20 °C and − 80 °C. Samples of both grinding conditions and storage temperatures were analyzed for tocopherol content after grinding (*t* = 0), after 1 week (*t* = 7) and after 4 weeks (*t* = 28). Each sample was measured in triplica for α-, β-, γ-, and δ-tocopherol as well α-tocotrienol. Three batches were produced in the same way over two months.

### 2.2 Determination of tocopherols and tocotrienols

The analysis was performed in an external accredited contracted laboratory. For the analysis, the method ASU L-4900-5 described in the official collection of analysis methods (Amtliche Sammlung von Untersuchungsvervahren) based on §35 of the Food Code (Lebensmittelgesetzbuch, DIN EN 12822:2014) was used. Unless otherwise specified, only analytically pure chemicals are used in the analysis. For the saponification, up to 20 g homogenized sample was placed in a 250 ml amber glass shaking funnel with 100 ml ethanol_BHT_ (96%), 1 g sodium ascorbate, 30 ml bidest water and 20 ml potassium hydroxide (60%). The beaker was introduced in a 85 °C hot water bath, heated till boiling under nitrogen atmosphere and kept simmering for 35 min. The saponification solution was transferred to a 500 ml amber glass shaking funnel, rinsed with 50 ml water and extracted with 100 ml n-hexane_BHT_ for 3 min on the horizontal shaker. The n-hexane phase was collected in a second beaker and the extraction repeated three times.

The combined hexane extracts were washed with three times 100 ml cold water and the wash water phase drained off until phase separation. The solution was filtered using sodium sulphate through a hydrophobic pleated filter into a 500 ml round-bottomed amber glass flask. The filtrate is concentrated on the rotary evaporator at 50 °C and evaporated to dryness under nitrogen. The sample extract was dissolved in n-hexane and membrane-filtered through a syringe filter into a sample vial.

The extract was analyzed by HPLC (HPLC Ultimate 3000, Thermo Scientific, USA) on a normal phase column (Si 250 × 4.6 mm, 5 μm, Inertsil) with attached pre-column (Guard Cartridge Kit Si, 4 × 3.0 mm). The eluent used was an isocratic solution of hexane, dioxane and 2-propanol. The flow was set to 1.3 ml/min. To the HPLC a fluorescent detector (FLD-3400, Thermo Scientific) was attached and fluorescent measured at 295 nm and 340 nm. Standards used for tocopherols were by Calbiochem (Merck, Darmstadt, Germany). The software Chromeleon 6.80 was used for quantification of the detected peaks. The level of quantification was set at 10 μg / 100 g, with an uncertainty of measurement of 10% and a recovery rate for α-tocopherol of 99.9%.

Statistical analysis of the experiments was performed by a multifactorial analysis of variance and Bonferroni adjustment with α < 0.05 using the statistical software package SPSS 21 (IBM, New York, USA).

## 3 Results and discussion

The found levels of α-, β-, γ-, δ-tocopherol and α-tocotrienol in blueberries and broccoli after homogenization and storage up to 28 days are listed in Table 1, Table 2. Please note that samples reported as “fresh” are always treated with liquid nitrogen before homogenization to prevent degradation of tocochromanols (see 2.1). As α-tocopherol is the most bioavailable tocochromanol, in the results and discussion section only changes in α-tocopherol are reported and discussed. Notably, of all the tocochromanols analyzed in this study, only α- and γ-tocopherol showed amounts above 0.1 mg/kg.

**Table 1:** Levels of tocochromanoles in blueberries with and without freeze drying during storage for up to 28 days at different temperatures (−20 °C, −80 °C).

α-Tocopherol					
		Homogenization condition			
Temperature	Storage	Freeze dried	St.dev.	Fresh	St.dev.
−20 °C	0 days	1.688	0.272	1.509	0.135
	7 days	1.651	0.266	1.622	0.203
	28 days	1.677	0.343	1.552	0.230
−80 °C	0 days	1.688	0.272	1.509	0.135
	7 days	1.707	0.259	1.637	0.130
	28 days	1.690	0.348	1.639	0.262

β-Tocopherol					
		Homogenization condition			
Temperature	Storage	Freeze dried	St.dev.	Fresh	St.dev.
−20 °C	0 days	0.014	0.002	0.014	0.003
	7 days	0.015	0.000	0.013	0.004
	28 days	0.014	0.002	0.014	0.004
−80 °C	0 days	0.014	0.002	0.014	0.003
	7 days	0.015	0.001	0.013	0.004
	28 days	0.014	0.004	0.014	0.004

γ-Tocopherol					
		Homogenization condition			
Temperature	Storage	Freeze dried	St.dev.	Fresh	St.dev.
−20 °C	0 days	0.518	0.073	0.509	0.102
	7 days	0.519	0.089	0.537	0.085
	28 days	0.503	0.052	0.531	0.063
−80 °C	0 days	0.518	0.073	0.509	0.102
	7 days	0.524	0.062	0.540	0.078
	28 days	0.503	0.078	0.534	0.071

δ-Tocopherol					
		Homogenization condition			
Temperature	Storage	Freeze dried	St.dev.	Fresh	St.dev.
−20 °C	0 days	0.016	0.004	0.004	0.006
	7 days	0.009	0.008	0.009	0.008
	28 days	0.009	0.003	0.008	0.007
−80 °C	0 days	0.016	0.004	0.004	0.006
	7 days	0.010	0.008	0.008	0.009
	28 days	0.010	0.003	0.009	0.008

α-Tocotrienol					
		Homogenization condition			
Temperature	Storage	Freeze dried	St.dev.	Fresh	St.dev.
−20 °C	0 days	0.016	0.004	0.010	0.001
	7 days	0.010	0.007	0.013	0.003
	28 days	0.009	0.003	0.012	0.002
−80 °C	0 days	0.016	0.004	0.010	0.001
	7 days	0.010	0.007	0.013	0.004
	28 days	0.010	0.003	0.012	0.003

**Table 2:** Levels of tocochromanoles in broccoli with and without freeze drying during storage for up to 28 days at different temperatures (−20 °C, −80 °C).

α-Tocopherol					
		Homogenization condition			
Temperature	Storage	Freeze dried	st.dev.	Fresh	st.dev.
−20 °C	0 days	1.632	0.132	1.303	0.067
	7 days	1.638	0.222	1.011	0.094
	28 days	1.613	0.245	0.810	0.139
−80 °C	0 days	1.632	0.132	1.303	0.067
	7 days	1.533	0.115	1.547	0.192
	28 days	1.613	0.348	1.314	0.086

β-Tocopherol					
		Homogenization condition			
Temperature	Storage	Freeze dried	st.dev.	Fresh	st.dev.
−20 °C	0 days	0.019	0.002	0.014	0.003
	7 days	0.018	0.004	0.015	0.002
	28 days	0.017	0.003	0.012	0.004
−80 °C	0 days	0.019	0.002	0.014	0.003
	7 days	0.016	0.002	0.015	0.005
	28 days	0.016	0.003	0.016	0.000

γ-Tocopherol					
		Homogenization condition			
Temperature	Storage	Freeze dried	st.dev.	Fresh	st.dev.
−20 °C	0 days	0.183	0.010	0.161	0.010
	7 days	0.186	0.039	0.210	0.131
	28 days	0.199	0.037	0.137	0.029
−80 °C	0 days	0.183	0.010	0.161	0.010
	7 days	0.154	0.010	0.179	0.035
	28 days	0.208	0.055	0.183	0.007

δ-Tocopherol					
		Homogenization condition			
Temperature	Storage	Freeze dried	st.dev.	Fresh	st.dev.
−20 °C	0 days	0.015	0.025	0.012	0.020
	7 days	0.028	0.024	0.009	0.015
	28 days	0.032	0.035	0.013	0.015
−80 °C	0 days	0.015	0.025	0.012	0.020
	7 days	0.024	0.022	0.021	0.036
	28 days	0.035	0.036	0.012	0.020

α-Tocotrienol					
		Homogenization condition			
Temperature	Storage	Freeze dried	st.dev.	Fresh	st.dev.
−20 °C	0 days	0.015	0.024	0.018	0.015
	7 days	0.028	0.024	0.016	0.010
	28 days	0.032	0.035	0.017	0.011
−80 °C	0 days	0.015	0.024	0.018	0.015
	7 days	0.024	0.022	0.027	0.030
	28 days	0.035	0.036	0.018	0.014

### 3.1 Blueberries

After homogenization of blueberry samples either with liquid nitrogen or after freeze-drying, the levels of α-tocopherol were measured (1.5 mg/100 g and 1.7 mg/100 g, respectively) (Table 1). In literature, differing α-tocopherol levels in blueberries were reported. Levels similar to the current research of 1.85 mg/100 g are stated by the German nutrient database (MRI (Max-Rubner-Institut), 2024). In contrast, a study by Bouzari, Holstege, and Barrett (2015) found approx. 10-times higher α-tocopherol concentrations of 13.93 and 20.84 mg/100 g fresh material in two blueberry batches. These batches were not treated with liquid nitrogen before homogenization. Differences might be explained by different growing conditions, varieties, degree of ripeness or different storage conditions.

**Fig. 1:**
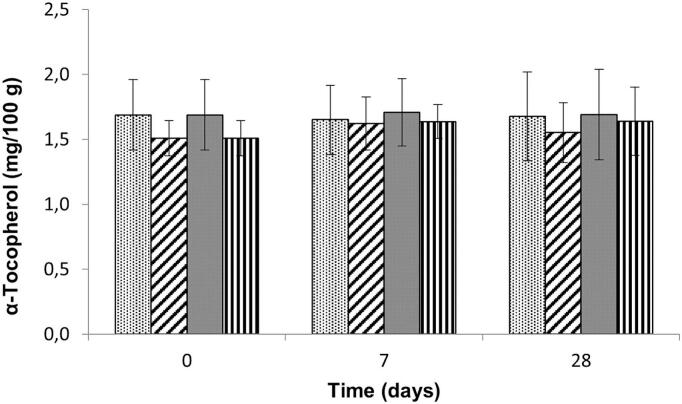
α-Tocopherol content (mg / 100 g) in blueberries after different storage temperatures (−20 °C, −80 °C) and homogenization conditions (with liquid nitrogen or freeze-dried). Black dots: freeze-dried and stored at −20 °C, diagonal stripes: homogenized with liquid nitrogen and stored at −20 °C, grey bars: freeze-dried and stored at −80 °C, vertical lines: homogenized with liquid nitrogen and stored at −80 °C.

In the current research, no significant differences could be found for homogenization procedure, storage temperature or storage over time up to 28 days or a combination of these factors (Fig. 1). Oxidative degradation of α-tocopherol during frozen storage, as described during storage of olive oil (Psomiadou & Tsimidou, 2002), could not be detected here. Similarly, no degradation of α-tocopherol could be found after storage at −20 °C or −80 °C for up to 180 days in human breast milk (Wei et al., 2018) or in the fruits of *opuntia ficus-indica* after 3 months at −20 °C (Bakar et al., 2020). In a study comparing fresh and frozen storage (−27.5 °C) of several fruits and vegetables, no significant differences between fresh and frozen stored blueberry samples up to 10 days could be observed (Bouzari et al., 2015). In contrast to the current research, both fresh and frozen stored samples showed increased α-tocopherol levels over the course of storage. It has to be noted that in none of the references in literature a treatment with liquid nitrogen before homogenization is mentioned.

### 3.2 Broccoli

The α-tocopherol content of broccoli samples after homogenization with liquid nitrogen or after freeze-drying was analyzed. In the initial material, levels of α-tocopherol varied from 1.30 mg/100 g to 1.62 mg/100 g for fresh (liquid nitrogen) and freeze-dried material, respectively (Table 2). Levels of initial materials found in broccoli in this research are comparable to literature. A study by Lee, Choi, Jeong, Lee, and Sung (2018) found α-tocopherol levels of 1.49 mg/100 g in broccoli, although treatment with liquid nitrogen is not mentioned. Other studies found levels of α-tocopherol in broccoli between 1.5 and 3.9 mg/100 g (Granado-Lorencio et al., 2007), average levels between 3.3 and 3.8 mg/100 g after freeze-drying (Knecht et al., 2015) and levels of 0.6 mg/100 g in the German nutrition database (MRI (Max-Rubner-Institut), 2024). Research conducted by Bernhardt and Schlich (2006) measured average α-tocopherol levels of 0.32 ± 0.05 mg/100 g fresh broccoli, without treatment of liquid nitrogen. These differences might be explained by different harvest years, different cultivars, different ripening degrees or different storage times and conditions.

**Fig. 2:**
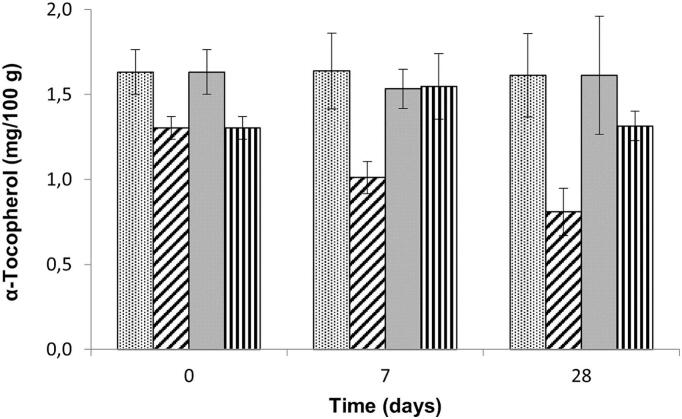
α-Tocopherol content (mg / 100 g) in Broccoli after different storage temperatures (−20 °C, −80 °C) and homogenization conditions (with liquid nitrogen or freeze-dried), expressed as mean ± sd). Black dots: freeze-dried and stored at −20 °C, diagonal stripes: homogenized with liquid nitrogen and stored at −20 °C, grey bars: freeze-dried and stored at −80 °C, vertical lines: homogenized with liquid nitrogen and stored at −80 °C. Bars marked with different letters (a, b, c) are significantly different (*p* ≤ 0.05).

Significant differences between homogenization conditions (freeze-dried/fresh with liquid nitrogen; *p* < 0.001) and storage temperature (−20 °C/−80 °C; *p* = 0.012) could be found (Fig. 2). Freeze-dried material showed no significant changes in α-tocopherol levels after storage at either −20 °C or − 80 °C for up to 28 days. Broccoli samples homogenized with liquid nitrogen and stored at −80 °C showed no change over time in levels of α-tocopherol, but had significant lower levels of α-tocopherol after storage at −20 °C for 7 or 28 days (*p* = 0.049 and *p* = 0.002, respectively). After storing fresh samples homogenized with liquid nitrogen for 7 days and 28 days at −20 °C, α-tocopherol levels decreased by approx. 23% and by approx. 38%, respectively (Table 1). In literature, blanched and frozen broccoli showed after 90 days of storage at −27.5 °C no significant differences (Bouzari et al., 2015). The blanching step before storage might have inactivated any relevant enzymes and protected the tocochromanols from enzymatic degradation (Lee et al., 2018). As enzymes were not inactivated in the current research, the detected reduction in α-tocopherol levels during storage at −20 °C might be caused by enzymatic degradation. An enzyme known to break down preferably α-tocopherol and which is also present in plants of the genus Brassica such as broccoli is tocopherol oxidase (Knecht et al., 2015). Tocochromanols are also known to degrade after photo-oxidative treatment in olive oil (Psomiadou & Tsimidou, 2002). The reduction of α-tocopherol concentration in broccoli stored at −20 °C might be explained by either enzymatic degradation or oxidative degradation.

The initial content of α-tocopherol was significantly different (*p* = 0.029) between the two homogenization procedures . After freeze-drying, α-tocopherol levels found were approx. 25% higher compared to the fresh initial material only treated with liquid nitrogen. α-Tocopherol content might be enhanced in freeze-dried material caused by better extractability from the matrix. Slightly related results could be found in drumstick leaves after freeze-drying (Sriwichai, Collin, Tranbarger, Berger, & Avallone, 2017). The bioaccessible α-tocopherol content increased at least 10-fold after freeze-drying at −40 °C and subsequent in vitro digestion. An increased α-tocopherol bioaccessibility could be also observed after sterilization, sun drying, oven drying and encapsulation. The found effect was explained with the destruction of the physicochemical barrier of cell walls and plastid substructure in processed leaves (Sriwichai et al., 2017). It was reported frequently that thermal treatment can enhance α-tocopherol levels in vegetables due to a softer structure leading to better extractability (Bernhardt & Schlich, 2006; Lee et al., 2018). Freeze-drying might have a similar effect on the extractability of broccoli by changing the cell structure.

A study by Knecht et al. (2015) reported pronouncedly higher α-tocopherol levels in baked than in fresh vegetables. The authors hypothesized that these differences are caused by enzymatic oxidation and would lead to an underestimation of vitamin levels in fresh material. Fresh vegetable samples were ground in preparation for the extraction at room temperature with addition of water and could lead to the contact between enzymes and substrates due to broken cell structures and cause degradation of α-tocopherol. In contrary, in the current study broccoli samples were immersed in liquid nitrogen and ground frozen to inhibit enzyme activity.

As the tocochromanol concentrations in the food before ingestion without a further processing step such as freeze-drying is of interest for the assessment of tocochromanol levels, fresh samples of the BfR MEAL Study for vitamin analysis should be frozen with liquid nitrogen. Further storage at −80 °C would be preferable, but even storage at −20 °C showed the same deviation from the initial fresh material as freeze-dried samples after 7 days. Although the analysis of vitamin relevant samples should be carried out as soon as possible, a storage time due to logistical problems till 7 days at −20 °C is justifiable.

The found differences in the degradation of tocopherol in blueberry and broccoli samples during frozen storage might be explained by either the different pH of blueberries (pH approx. 3.6 (Ścibisz & Ziarno, 2023)) and broccoli (pH 5.6 to 6.4 (Hanschen et al., 2017)), leading to different inhibition of enzymes, or the different cell structure of the material. In literature, after addition of acetic acid till pH of 4.24 and 4.28 to broccoli samples, higher tococpherol concentrations could be found (Knecht et al., 2015). This was explained by inhibiting the tocopherol degrading enzyme. In the current research, significant differences could be found between fresh and freeze-dried broccoli samples while no differences could be found between fresh and freeze-dried blueberry samples. The softer cell structure of blueberries might be easily destroyed and lead to similar extraction results in fresh and freeze-dried samples, while broccoli has a stronger cell structure and freeze-drying might lead to more damage to cell structure and consequently to a higher extractability of tocochromanols.

## 4 Summary and conclusion

Levels of α-tocopherol in blueberries were similar after storage at −20 °C or − 80 °C in fresh and freeze-dried homogenized samples up to 28 days. In contrast, in broccoli samples, levels of α-tocopherol were higher after freeze-drying compared to fresh broccoli samples. This might be caused by a higher extractability of α-tocopherol due to damage of cell structure. Fresh broccoli samples showed a degradation of α-tocopherol levels during storage at −20 °C after 7 and 28 days. These findings suggested an influence of pre-storage treatment and storage temperature on the measured α-tocopherol content, which appears to correlate with the pH of the food. As the pre-analytical treatment may reduce or increase the subsequently measured levels, the treatment of the samples must be carefully assigned for future studies. If freeze-drying is applied to the initial material, the found levels might be overestimated, while levels in samples stored at −20 °C might be underestimated.

For the BfR MEAL study the levels of α-tocopherol in the eaten food should be represented, without any further processing step.. Fresh broccoli samples stored for 7 days showed the same deviation from the initial fresh material than freeze-dried broccoli. This led to the conclusion that the storage of the fresh food samples relevant for vitamins at −20 °C and no longer than 7 days after homogenization prior to analysis is acceptable.

## CRediT authorship contribution statement

**Irmela Sarvan:** Writing – original draft, Visualization, Validation, Project administration, Methodology, Investigation, Formal analysis, Conceptualization. **Anton Jürgensen:** Writing – review & editing, Validation. **Matthias Greiner:** Writing – review & editing, Supervision. **Oliver Lindtner:** Writing – review & editing, Project administration, Methodology, Funding acquisition, Conceptualization.

## Declaration of competing interest

There has been no conflict of interest for any author.

## Data Availability

Data will be made available on request.
